# Phase 1 trial to model primary, secondary, and tertiary dengue using a monovalent vaccine

**DOI:** 10.1186/s12879-023-08299-5

**Published:** 2023-05-23

**Authors:** Camila D. Odio, Kelsey E. Lowman, Melissa Law, Rosemary A. Aogo, Sally Hunsberger, Brad J. Wood, Michael Kassin, Elliot Levy, Viviane Callier, Saba Firdous, Chloe M. Hasund, Charlie Voirin, Robbie Kattappuram, Christina Yek, Jessica Manning, Anna Durbin, Stephen S. Whitehead, Leah C. Katzelnick

**Affiliations:** 1 Viral Epidemiology and Immunity Unit, Laboratory of Infectious Diseases, National Institute of Allergy and Infectious Diseases, National Institutes of Health, Bethesda, MD USA; 2 Laboratory of Viral Zoonotics, Department of Veterinary Medicine, University of Cambridge, Cambridge, UK; 3 Division of Clinical Research, Biostatistics Research Branch, National Institute of Allergy and Infectious Diseases, National Institutes of Health, Bethesda, MD USA; 4 Interventional Radiology and Center for Interventional Oncology, NIH Clinical Center and National Cancer Institute, National Institutes of Health, Bethesda, MD USA; 5 Clinical Monitoring Research Program Directorate, Frederick National Laboratory for Cancer Research, Frederick, USA; 6 Department of Pharmacy, NIH Clinical Center, National Institutes of Health, Bethesda, MD USA; 7 International Center of Excellence in Research, National Institute of Allergy and Infectious Diseases, National Institutes of Health, Phnom Penh, Cambodia; 8 Laboratory of Malaria and Vector Research, National Institute of Allergy and Infectious Diseases, National Institutes of Health, Bethesda, MD USA; 9 Department of International Health, Johns Hopkins Bloomberg School of Public Health, Baltimore, MD USA; 10 Arbovirus Vaccine Research Section, Laboratory of Viral Diseases, National Institute of Allergy and Infectious Diseases, National Institutes of Health, Bethesda, MD USA

**Keywords:** Dengue, Vaccine, Live-attenuated, Primary, Secondary, Tertiary, Natural infection, Lymph node aspirates, Germinal center

## Abstract

**Background:**

The four co-circulating and immunologically interactive dengue virus serotypes (DENV1-4) pose a unique challenge to vaccine design because sub-protective immunity can increase the risk of severe dengue disease. Existing dengue vaccines have lower efficacy in DENV seronegative individuals but higher efficacy in DENV exposed individuals. There is an urgent need to identify immunological measures that are strongly associated with protection against viral replication and disease following sequential exposure to distinct serotypes.

**Methods/Design:**

This is a phase 1 trial wherein healthy adults with neutralizing antibodies to zero (seronegative), one non-DENV3 (heterotypic), or more than one (polytypic) DENV serotype will be vaccinated with the live attenuated DENV3 monovalent vaccine rDEN3Δ30/31-7164. We will examine how pre-vaccine host immunity influences the safety and immunogenicity of DENV3 vaccination in a non-endemic population. We hypothesize that the vaccine will be safe and well tolerated, and all groups will have a significant increase in the DENV1-4 neutralizing antibody geometric mean titer between days 0 and 28. Compared to the seronegative group, the polytypic group will have lower mean peak vaccine viremia, due to protection conferred by prior DENV exposure, while the heterotypic group will have higher mean peak viremia, due to mild enhancement. Secondary and exploratory endpoints include characterizing serological, innate, and adaptive cell responses; evaluating proviral or antiviral contributions of DENV-infected cells; and immunologically profiling the transcriptome, surface proteins, and B and T cell receptor sequences and affinities of single cells in both peripheral blood and draining lymph nodes sampled via serial image-guided fine needle aspiration.

**Discussion:**

This trial will compare the immune responses after primary, secondary, and tertiary DENV exposure in naturally infected humans living in non-endemic areas. By evaluating dengue vaccines in a new population and modeling the induction of cross-serotypic immunity, this work may inform vaccine evaluation and broaden potential target populations.

**Trial Registration:**

NCT05691530 registered on January 20, 2023.

## Background

Dengue is the most prevalent vector-borne viral disease globally, causing between 50 and 100 million cases annually [1]. In 2017, more than two million disability adjusted life years were attributed to dengue, and it is one of the few communicable diseases with an increasing global burden [2, 3]. Despite its high morbidity, a universally effective vaccine against dengue has been elusive [4]. Dengue is caused by any of the four dengue virus serotypes (DENV1-4). A first DENV infection is generally not severe and is thought to induce long-lived protection against reinfection with that serotype. In contrast, risk of dengue hemorrhagic fever/dengue shock syndrome is 24 times greater during secondary DENV infection with a different serotype as compared to primary infection [5]. A longer interval between DENV infections also correlates with severe disease [6]. Severe dengue may be associated with antibody-dependent enhancement, where low levels of cross-reactive antibodies induced by the primary infection promote internalization and replication of the secondary infecting virus in cells with Fcγ receptors, resulting in earlier and higher peak viremia and a dysregulated immunological response [7]. Higher mean peak viremia and severe dengue are also associated with delayed CD8^+^ T cell responses [8].

To avoid inducing antibody-dependent enhancement, the three leading vaccine candidates - Dengvaxia, TAK-003, and TV003 – are live-attenuated, tetravalent, and aim to induce specific immunity against each serotype simultaneously. TV003 phase 1/2 trials indicate that it induces a tetravalent neutralizing antibody response in about two-thirds of subjects [9–11]. Phase 3 efficacy trials are ongoing, but initial 2-year follow-up data indicate 90% efficacy against DENV1 regardless of prior serostatus, 84% efficacy against DENV2 in DENV-seropositive individuals, and 58% efficacy against DENV2 in DENV-seronegative individuals [12]. There were insufficient DENV3 and DENV4 cases to report efficacy against these serotypes, and these data are still being collected. Surprisingly, although Dengvaxia and TAK-003 induce antibodies that neutralized all four DENV serotypes in vitro, vaccine efficacy varied by strain, and seroconversion alone did not predict protection [13]. While Dengvaxia induces strong protection against DENV4 and TAK-003 against DENV2, neither vaccine provides full protection against other serotypes, and both have significant waning of efficacy over time [14–18]. Most concerningly, Dengvaxia, which was licensed in 20 countries and introduced in mass vaccination programs in the Philippines and Brazil, significantly increases risk of hospitalized dengue in DENV seronegative individuals.

Second heterotypic infection has the highest risk of severe dengue, but third and fourth infections are typically less severe [19]. It is hypothesized that secondary DENV exposure induces broadly neutralizing antibodies through maturation of the low-affinity, cross-reactive B cells induced by primary exposure [20–22]. Sequential infection is also associated with an evolution of the T cell response from targeting serotype-specific epitopes to conserved regions [23]. Notably, both Dengvaxia and TAK-003 have higher efficacy in DENV seropositive than seronegative individuals, suggesting that prior DENV infection also can safely improve the immunogenicity and protection conferred by the vaccination. Heterologous sequential immunization may induce potent broadly neutralizing antibodies and T cells by focusing cellular and humoral responses on protective epitopes conserved across serotypes. One pre-clinical study of dengue DNA vaccines in mice reported that compared to repeat tetravalent vaccination, sequential monovalent vaccination with distinct serotypes induced stronger cellular and humoral responses to both exposed and nonexposed serotypes [24]. In the only human sequential immunization trial with monovalent live-attenuated dengue vaccines, earlier onset and higher mean peak viremia as well as higher rash frequency, were the only adverse effects observed after secondary heterotypic versus primary DENV2 vaccination [25]. Initial vaccination resulted in potent type-specific and weak cross-reactive antibodies, while secondary vaccination induced effective neutralizing antibodies against both exposed and nonexposed serotypes. This study provides proof-of-concept for the immunological insights to be learned from safe human models of sequential DENV exposure.

Building on previous work, we aim to evaluate how distinct DENV immunity profiles impact the viral, innate, cellular, and humoral responses to a controlled DENV exposure. Additionally, we will evaluate the paradigm of image guided serial nodal immune characterization as a key element of model design. By accounting for other variables, our study has the potential to identify novel biomarkers associated with viral replication and the process by which sequential exposure induces broadly protective dengue immunity.

### Vaccine choice and previous studies

#### Choice of rDEN3Δ30/31-7164

The monovalent rDEN3Δ30/31-7164 is one of the four viral components of the tetravalent dengue vaccine candidate, TV003, developed by NIAID/NIH. It is derived from the wild-type DENV-3 Sleman/78 virus with 30 and 31 non-contiguous nucleotide deletions in the 3’ untranslated region (UTR) for attenuation, and a Vero cell adaptation mutation (nucleotide position 7164, corresponding to amino acid 115 in NS4B Val→Ala) [26, 27]. Of the four monovalent vaccine strains, rDEN3Δ30/31-7164 had one of the lowest mean peak viremia titers and standard errors, suggesting intrinsically consistent replication, and viremia was less frequent in DENV seropositive versus seronegative individuals in Brazil [11, 28]. Thus, we expect that rDEN3Δ30/31-7164 inoculation will maintain a relatively low mean peak viremia titer, and that differences in viremia titer between groups can likely be attributed to host immunity rather than vaccine strain variability.

DENV3 is a clinically relevant and immunologically unique serotype, and the use of rDEN3Δ30/31-7164 in this trial will expand on previous work. Specifically, low levels of pre-existing anti-DENV antibodies enhance DENV3 while high levels are protective, making our trial an ideal model of both presentations [29]. This contrasts with DENV1, where cross-reactive immunity protects against DENV1 secondary disease, and DENV2, which is enhanced across a broad range of pre-existing antibody titers [30]. In clinical trials, the TAK-003 vaccine failed to protect seronegative subjects against DENV3 symptomatic and hospitalized dengue disease [19, 31, 32]. This failure may be related to the unsuccessful replication of the DENV3 component of TAK-003, though the vaccine did protect seronegative subjects against DENV1 despite no detected replication of this component [33]. Thus, the basis for the failure of TAK-003 to protect against DENV3 is not completely understood. Despite this knowledge gap, the only sequential monovalent vaccination trial performed in humans utilized DENV1, 2, and 4, while monovalent rDEN3Δ30/31-7164 vaccination was only assessed in flavivirus-seronegative individuals [25, 30, 34]. The Walter Reed Army Institute of Research has performed two trials using a different challenge strain of DENV3, testing a total of seven seronegative individuals and ten individuals who had been immunized with a tetravalent dengue vaccine candidate, but neither trial included individuals with previous natural infection [35, 36]. By comparing responses in the polytypic versus heterotypic groups, our study may identify relevant markers and/or drivers of serotype-specific immunity versus mild enhancement. In sum, scrutinizing the responses to a live attenuated full-length DENV3 will help address clinically and immunologically relevant questions.

#### Pre-clinical and clinical data for rDEN3Δ30/31-7164

Compared to its parent strain, rDEN3Δ30/31-7164 has restricted replication in juvenile rhesus monkeys, a model of severe combined immunodeficiency (SCID) mice, and mosquitos [26]. rDEN3Δ30/31-7164 has been evaluated in one phase 1 clinical trial, and TV003 has been evaluated in multiple phase 1 and 2 trials [9–11, 28, 37, 38], with full phase 3 trial data pending (NCT02406729). Although rDEN3Δ30/31-7164 monovalent vaccination has never been tested in flavivirus exposed individuals, the virus has been administered to immune individuals as part of several tetravalent formulations with excellent safety profiles. In all three phase 1 trials – monovalent rDEN3Δ30/31-7164 (n = 50), TV003 in flavivirus seronegative participants (n = 60), and TV003 in flavivirus-seropositive participants (n = 29 with previous yellow fever virus vaccine, n = 11 with monovalent DENV vaccine, n = 8 with either West Nile virus, St. Louis encephalitis, or Japanese encephalitis virus immunity) – only rash was more frequent in the vaccine versus placebo groups [9, 10, 28]. In the study of TV003 in flavivirus-seropositive participants, where most individuals had yellow fever virus immunity rather than DENV immunity (60% vs. 23%), rDEN3Δ30/31-7164 was the only serotype to have higher viremia titer in the flavivirus-seropositive versus the seronegative group (mean titer: 0.97 vs. 0.60 log_10_ PFU/mL, p = 0.04; maximum titer: 2.4 vs. 1.2 log_10_ PFU/mL). In Brazil, vaccine viremia was more frequent in the DENV seronegative group versus the DENV seropositive group, and in India, viremia was not observed in any participant, all of whom were DENV-seropositive. Thus, the type of underlying flavivirus immunity and the time from last exposure likely influence viremia frequency and peak titers. However, regardless of DENV serostatus, the peak rDEN3Δ30/31-7164 viremia (10^2.4^ PFU/mL) remained orders of magnitude lower than that required for mosquito transmission (10^5^ infectious units/mL) or that observed in symptomatic (10^6^ infectious units/mL) and severe dengue (10^8^ infectious units/mL) [25, 27]. Thus, rDEN3Δ30/31-7164 is a safe and relevant model for examining enhancing and protective immune responses.

## Methods/Design

### Study objectives and endpoints

**Fig. 1:**
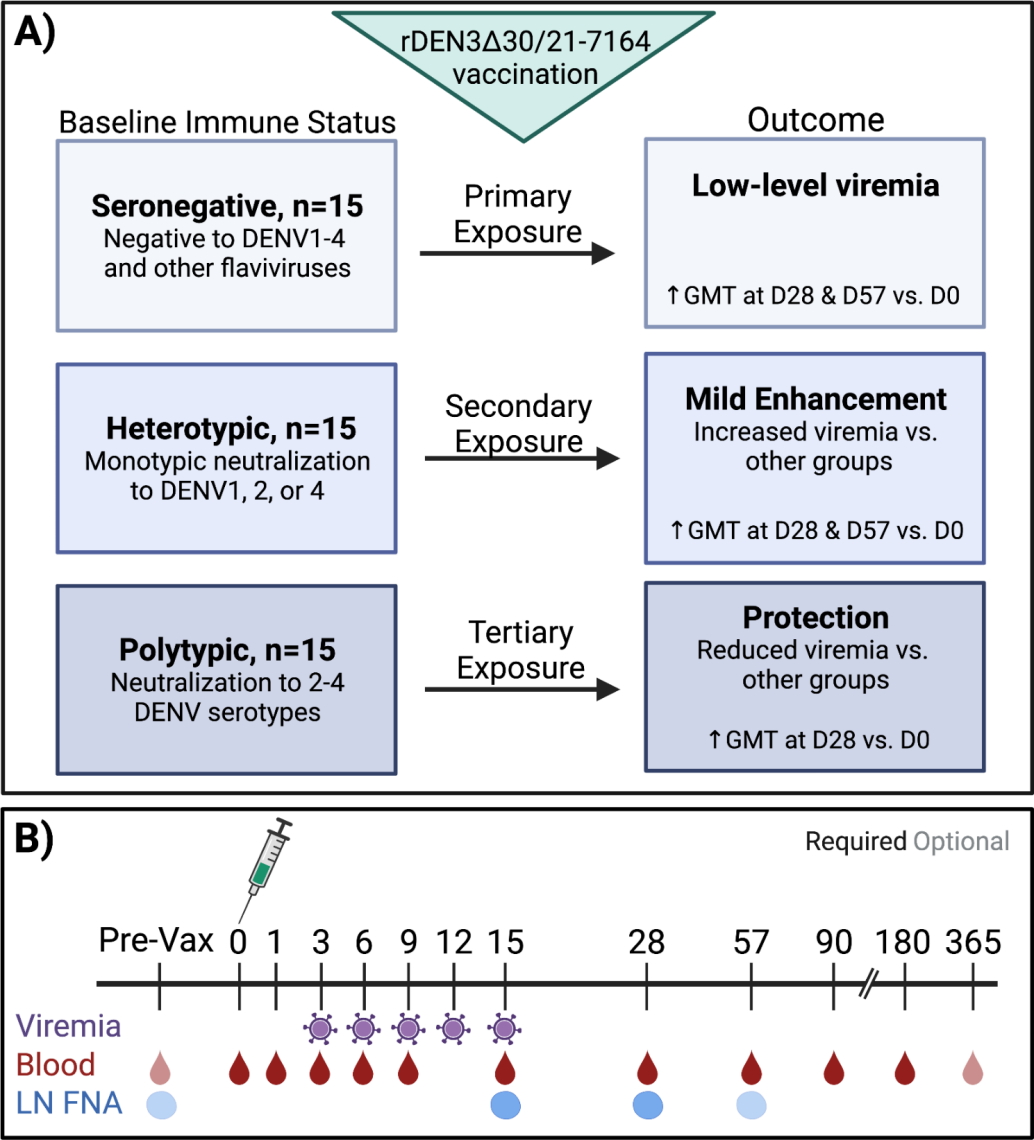
Trial design. Baseline immune status of each group and expected outcome after DENV3 vaccination (**A**). The trial includes optional pre-vaccination consent, blood draw, and lymph node aspiration, which can occur between days − 59 and 0 (**B**). Vaccination and subsequent blood draws, viremia assessments, and lymph node aspirations are outlined. Created with BioRender.com.

This is a phase 1, partially blinded study with the primary objective to evaluate how DENV immune status (seronegative, non-DENV3 monotypic, or polytypic) modulates safety and protection against immunization with live-attenuated monovalent vaccine rDEN3Δ30/31-7164 (Fig. 1A). The primary study endpoints are: (i) the frequency and severity of local and systemic reactogenicity signs and symptoms during the 28 day period after each vaccination, unexpected adverse events (AEs) up to 28 days after each vaccination, and serious adverse events (SAEs) through day 180; (ii) change in DENV1-4 neutralizing antibody geometric mean titer (GMT) between days 0 and 28; and (iii) mean peak viremia among groups as measured by viral quantitative reverse transcription polymerase chain reaction (qRT-PCR) between days 3 and 15 (Table 1). We hypothesize that the vaccine will be safe and well tolerated, and the groups will have evidence of infection with the vaccine strain as indicated by a statistically significant increase in the DENV1-4 neutralizing antibody GMT between days 0 and 28 and/or measurable viremia between days 3 and 15. The polytypic group will have lower mean peak viremia than the seronegative group, indicating protection resulting from prior exposures, while the heterotypic group will have higher mean peak viremia due to mild enhancement of vaccine replication. The secondary objective is to further evaluate how DENV immune status impacts the immunogenicity of the vaccine by examining changes in neutralizing antibody GMT at day 57 and the CD8^+^ T-cell response at day 15 among groups. Exploratory endpoints will evaluate innate and adaptive cell responses over the course of infection, cellular activity of actively DENV-infected cells, and the phenotypic, transcriptional, and molecular profiles of immune cells and their receptors in peripheral blood and lymph nodes by single cell sequencing. Repetitive lymph node sampling will enable exploration into the cellular dynamics of germinal center reactions through longitudinal assessment of B cell recruitment and maturation, Tfh cell signaling and presentation, and the frequency of dengue-infected cells.

**Table 1:** Study Objectives and Endpoints (Selected)

OBJECTIVES	ENDPOINTS	
Primary		
Evaluate the safety of monovalent DENV3 vaccination in those with distinct natural DENV infection histories living in non-endemic areas, and how prior DENV immunity influences protection against vaccine strain infection evaluated by the change in GMT and mean peak viremia.	The frequency and severity of local and systemic reactogenicity signs and symptoms during the 28 day period after each vaccination, unexpected AEs up to 28 days after each vaccination, and SAEs through day 180.	
	Change in the DENV1-4 neutralizing antibody GMT between days 0 and 28.	
	Mean peak viremia among groups as measured by viral qRT-PCR between days 3 and 15.	
Secondary		
Further evaluate how DENV infection history impacts the immunogenicity of the vaccine.	Change in DENV1-4 neutralizing antibody GMT between days 0 and 57.	
	DENV1-4 neutralizing antibody GMT at days 0, 28, and 57.	
	Magnitude of the CD8^+^ T-cell response at day 15 among groups as measured by AIM assays.	
Tertiary/Exploratory		
Further evaluate how DENV infection history impacts the immune response to rDEN3Δ30/31-7164 (all potential timepoints are listed; exploratory analyses will be conducted at selective timepoints depending on the results of primary and secondary endpoints).	Change in DENV3 neutralizing antibody titer between day 0 and peak titer (measured at days 28, 57, or 90).	
	Characterize the broadly neutralizing antibodies observed in each group at screening and may be performed at days 15, 28, 57, 90, 180, and 365 after vaccination using ELISAs and Blockade-of-Binding assays with previously isolated potently neutralizing antibodies.	
	Assess whether ADE assays are associated with increased rDEN3Δ30/31-7164 viremia.	
	Immunophenotyping at days 0, 1, 3, 6, 9, 15, and 28 post-vaccination in each group.	
	Characterize the phenotype, frequency, and magnitude of CD4^+^ and CD8^+^ T cells and T follicular helper (Tfh) cells in each group. These analyses may be performed at days 0, 3, 6, 9, 15, 28, 57, 90, and 180 using AIM assays.	
	Compare rash frequency among groups during the first 28 days post-vaccination.	
	Characterize the transcriptome and cell surface proteins in each group using single-cell RNA sequencing (RNAseq) and cellular indexing of transcriptomes and epitopes by sequencing (CITE-seq). These analyses may be performed at days 0, 1, 3, 6, 9, 15, 28, and 57.	
	Characterize serum cytokine responses using Luminex assays.	
Evaluate how DENV infection history impacts the immune response to rDEN3Δ30/31-7164 in the LN.	Characterize the B and T cell phenotypes in peripheral blood versus the lymph node in each group by surface protein staining, AIM assays, and single-cell RNA-seq, BCR and TCR sequencing, and CITE-seq pre-vaccine and at days 15, 28, and 57.	
	Assess the nucleotide mutation frequencies in the immunoglobulin heavy chain (IGHV) genes of germinal center B cells at days 15, 28, and 57 in each group using BCR-seq.	
	Assess whether the nucleotide mutation frequencies in the IGHV genes of germinal center B cells at days 15, 28, and 57 are associated with the presence of potent, cross-serotypic antibodies.	
Evaluate the cells involved in viral replication.	Assess PBMCs for DENV viral protein expression using intracellular staining for non-structural protein 3 (NS3).	

ADE, antibody dependent enhancement; AE, adverse events; AIM, activation induced marker assays; GMT, geometric mean titer; SAE, severe adverse events

### Study design and participants

**Fig. 2:**
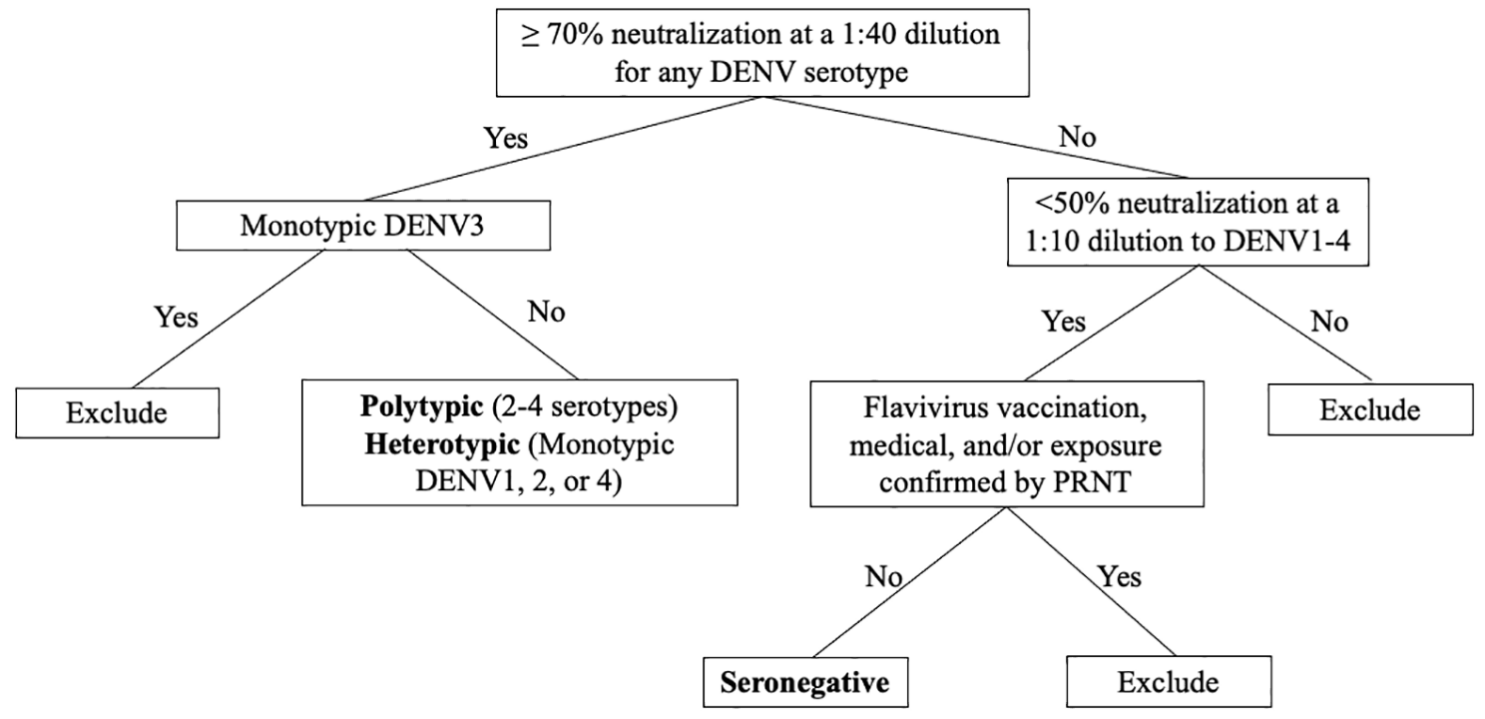
Schema for group determination. All individuals will be assessed for dengue immunity by plaque reduction neutralization assays. Individuals will be considered immune if they have ≥ 70% reduction in infectious plaques at a 1:40 dilution. Those with monotypic immunity to DENV1, DENV2, or DENV4 (**heterotypic** to vaccine) and those with immunity to ≥ 2 serotypes, which can include DENV3 (**polytypic**), will be eligible. Individuals who do not meet the immunity threshold must have < 50% reduction in infectious plaques at 1:10 dilution against all serotypes to be included in the DENV **seronegative** group. Potential exposure to other flaviviruses as suggested by travel and/or vaccine history will trigger confirmatory antibody testing for the virus of interest. All seronegative individuals must also be flavivirus naïve.

The study will be conducted at the National Institutes of Health Clinical Center in Bethesda, Maryland. Approximately 200 individuals aged 18 to 59 will be screened to determine if they meet eligibility criteria, and 45 will be enrolled (Table 2). Serotype-specific DENV immunity will be determined by the plaque (immunofocus) reduction neutralization test (PRNT). Because anti-DENV antibody waning can occur over time, we have a high threshold for DENV-immune and a low threshold for DENV-seronegative (Fig. 2). All individuals will complete a survey regarding previous DENV and flavivirus exposure. This information will be used to confirm naïve status in the DENV-seronegative individuals. Uncertainty regarding possible exposures to other flaviviruses will trigger confirmatory testing via PRNT against the virus of interest (e.g. Japanese encephalitis virus, West Nile virus, Zika virus, yellow fever virus). Fifteen participants who meet criteria will be enrolled in each of the three study groups: flavivirus-seronegative, non-DENV3 monotypic (heterotypic to the vaccine serotype), and polytypic.

**Table 2:** Eligibility Criteria

Inclusion criteria	Exclusion criteria
1. Aged 18 to 59 years. 2. In good general health as evidenced by medical history, physical examination, and laboratory screening results^a^. 3. Willing to allow storage of samples and data for future research. 4. Willing to forgo receipt of any vaccine in the 28 days preceding the vaccine or in the 28 days following the dose of vaccine. For participants opting for LN FNA on day 57, they must be willing to forgo any vaccine through final LN FNA. 5. For individuals who can become pregnant: use of at least one method of highly effective contraception^b^ from the invitation to participate in the trial through day 28 after vaccination. 6. Able to provide informed consent. 7. Willing to adhere to lifestyle considerations^c^ for the duration of the study. 8. Willing to avoid travel to a dengue-endemic areas as defined by the Centers for Disease Control and Prevention (CDC) for the duration of their participation in the study. 9. Absolute neutrophil count (ANC) > 750 cells/µL. 10. Creatinine < 1.5 mg/dL. 11. ALT < 1.25 × upper limit of normal. 12. Flavivirus-naïve or serologic evidence of either one previous DENV1, 2, or 4 infection or infection with at least two different serotypes. 13. Agree to avoid participation in other clinical studies requiring investigational interventions for the duration of this study (180 days). 14. Agree to avoid blood and plasma donation outside this study through day 28.	1. Pregnant at screening. 2. History of or positive test result for HIV, hepatitis B, or hepatitis C. 3. History of previous DENV vaccination 4. Any medical, psychiatric, or social condition that, in the judgement of the investigator, is a contraindication to protocol participation. 5. Has any of the following: a) More than 10 days of systemic immunosuppressive medications (≥ 10 mg prednisone dose or its equivalent) or cytotoxic medication within the 30 days prior to vaccination or immunomodulating therapy^d^ within 180 days prior to vaccination. b) Received blood products, including immunoglobulin products, within 120 days prior to vaccination. c) History of serious reactions to vaccines. d) Hereditary, acquired, or idiopathic forms of angioedema. e) Idiopathic urticaria within the past year. f) Asthma that is not well controlled or required emergency care, urgent care, hospitalization, or intubation during the past two years or that requires the use of oral or intravenous steroids. g) Type 1 or type 2 diabetes mellitus that is not well controlled (hemoglobin A1c > 8). h) Clinically significant autoimmune disease or immunodeficiency. i) Blood pressure ≥ 180/110 (stage 3 hypertension) on at least 2 measures. j) Documented diagnosis of a bleeding disorder (e.g., factor deficiency, coagulopathy, or platelet disorder requiring special precautions). k) Significant bruising or bleeding difficulties with subcutaneous injections or blood draws. l) Malignancy that is active or treated malignancy for which there is no reasonable assurance of sustained cure, or malignancy that is likely to recur during the study period. m) Asplenia or functional asplenia. n) History of alcohol or drug abuse or addiction and/or positive drug screen with substances other than marijuana.

^a^Laboratory screening will occur within 60 days of vaccination and values from the screening visit only will determine eligibility, except for pregnancy testing, which will need to be negative on day 0 as well

^b^Contraception options are: intrauterine device or equivalent; hormonal contraceptive; condom, diaphragm, or cervical cap plus spermicide; a stable, long-term monogamous relationship with a partner who does not pose any potential pregnancy risk (e.g., has undergone a vasectomy at least 6 months prior to vaccination or is of the same sex as the participant); a hysterectomy and/or a bilateral tubal ligation, bilateral oophorectomy; or postmenopausal status defined as age ³ 45 years and at least 1 year since last menstrual period

^c^Lifestyle considerations are: abstain from excessive alcohol intake for 24 h before blood collection (> 1 drink for women, > 2 drinks for men), and abstain from strenuous exercise for 12 h before each blood collection

^d^Immunomodulating therapies include ≥ 10 mg prednisone equivalent per day planned for ≥ 14 days, cytotoxic drugs (e.g., methotrexate, cyclophosphamide, doxorubicin, vincristine), alemtuzumab, azathioprine, cyclosporine and tacrolimus, mycophenolate mofetil, rapamycin, anti-lymphocyte serum and anti-thymocyte globulins (e.g., ATG, OKT3), rituximab, anti-TNF-α inhibitors (e.g., infliximab, adalimumab, etanercept), anti-IL-2 receptor monoclonal antibodies (e.g., basiliximab), purine analog therapy (e.g., cladribine, pentostatin)

Each participant will receive one dose of rDEN3Δ30/31-7164 on study day 0 and will have clinical evaluations and AE assessments on days 6, 9, 15, and 28 in the first month (Table 3). These days were chosen to maximize the opportunity to observe rash, which is the AE that is most likely to differ among groups. Previous trials have indicated that rash onset after TV003 occurs from days 7 to 15, and typically lasts 5 to 10 days. No safety signals were observed prior to day 6 or between days 15 and 28 (clinical protocol from [28] supplementary material). Blood draws for viremia, collection of PBMCs, clinical monitoring labs, and/or antibody titers will occur on days 0, 1, 3, 6, 9, 12, 15, 28, 57, 90, and 180, with an optional draw on day 365 (Fig. 1B). Assessments for solicited injection site and systemic reactions related to vaccination will occur through day 28. Unexpected AEs and SAEs that are possibly, probably, or definitely related to rDEN3Δ30/31-7164 vaccination will be collected through day 180, the last required clinical visit. Participants can choose to attend an optional day 365 visit, which will allow us to examine the longevity of responses.

**Table 3:** Schedule of Activities

	Screen	Optional^a^	Schedule of Activities															Optional^b^
**Visit Number**	Screen		SC^c^	01	02	03	04	05	06	07	SC^c^	08	SC^c^	09	SC^c^	10	11	12
**Day of Study** ^**d**^			−59 to 0	D0	D1	D3	D6	D9	D12	D15	D16	D28	D29	D57	D58	D90	D180	D365
**Visit Window (days)**	−60 to − 1	−59 to 0	+ 2	0	0	± 2	± 2	± 2	± 2	± 4	+ 1	± 4	+ 1	+ 12	+ 1	± 10	± 15	± 15
**Clinical Evaluations/ Procedures**				**Active Phase**										**Follow-up Phase**				
DENV3 vaccine screening consent	X																	
DENV3 vaccine full study informed consent^e^		X		X														
Demographics	X																	
Medical history/focused physical examination	X	X		X			X	X		X		X		X			X	X
Vital signs^f^	X	X		X			X	X		X		X		X			X	X
Height/weight	X																	
Review of health history	X			X						X		X		X			X	X
Travel history assessment	X^g^			X^h^														
Concomitant medication review	X	X		X			X	X		X		X		X			X	X
Pregnancy prevention counseling	X			X														
Communication for any aspirate-related AEs			X								X		X		X			
Administer rDEN3Δ30/31-7164 vaccine^i^				X														
AE assessment^j^				X			X	X		X		X		X			X	X
Optional lymph node fine needle aspiration^k^		X								X		X		X				
DENV3 vaccine screening consent	X																	
Optional lymph node fine needle aspiration^k^		X								X		X		X				
Optional photos of rash, if present							X	X		X		X		X				
CBC with differential	X^l^	X		X	X	X	X	X	X	X		X		X				
Acute care and hepatic panel	X	X		X				X	X	X		X		X				
PT/PTT/INR	X	X		X				X	X	X		X		X				
HBsAg, anti-HCV antibody	X																	
Anti-HIV 1/2 antibody/Ag	X																	
HLA typing				X														
Human chorionic gonadotropin, pregnancy^m^	X	X		X						X		X		X				
Drug abuse screen, urine	X																	
Research blood (flavivirus antibodies, neutralizing antibody assays, serum storage, viral qRT-PCR, and culture)^n^	X			X	X	X	X	X	X	X		X		X		X	X	X
Research blood (T and B cell assays; plasma, PBMC storage)		X		X	X	X	X	X		X		X		X		X	X	X

**Abbreviations**: AE, adverse event; CBC, complete blood count; DENV, dengue virus; HbA1c, hemoglobin A1c; HBsAg, hepatitis B virus surface antigen; HCV, hepatitis C virus; HIV, human immunodeficiency virus; INR, international normalized ratio; OTC, over the counter; PBMC, peripheral blood mononuclear cells; PT, prothrombin time; PTT, partial thromboplastin time; qRT-PCR, quantitative reverse transcription polymerase chain reaction; SC, secure communication; X, to be performed

^a^Optional visit for lymph node aspiration to obtain baseline cell populations

^b^Optional blood draw on day 365 to assess antibody waning

^c^All patients who undergo lymph node aspiration will receive a follow-up call or secure electronic communication (per participant preference) within 24 to 48 h to assess for any procedure-related AEs.

^d^An early termination visit will be performed if the participant decides to discontinue participation or is withdrawn from the study prior to day 180. It will include a targeted history, focused physical exam, and assessment of any unresolved AEs, including abnormal laboratory results

^e^Written informed consent for screening must be obtained prior to initiation of screening procedures, and written informed consent for the DENV3 vaccination protocol must be obtained at the optional baseline lymph node aspiration day or day 0 prior to the initiation of any study procedures

^f^Complete post-vaccination evaluation (temperature, blood pressure, pulse, respiratory rate, and injection site assessment) will be performed 30 to 60 min post-vaccination. For those undergoing lymph node aspiration, vital signs will also be assessed per Clinical Center and interventional radiology guidelines

^g^Short travel history completed at pre-screening will only be reviewed by unblinded statistician to assist with screening target groups

^h^Post-enrollment travel history assessment can be completed any time between days 0 and 57. Study staff will not review the assessment until after study unblinding

^i^For participants who can get pregnant, confirm that pregnancy test is negative on day 0, and for all participants, review safety labs from screening day (CBC, acute care, hepatic panels) prior to vaccine administration. Day 0 safety labs are for baseline only and will not determine eligibility

^j^Day 0 evaluations, prior to the first vaccine dose, are the baseline for assessing subsequent AEs.

^k^Lymph fluid will be collected, with minimal blood loss anticipated (< 5 mL). Participants will be monitored post-procedure until the responsible provider deems they are safe to leave the Clinical Center. Lymph node aspirations on days 15 and 28 are opt-out procedures, and pre-vaccine and day 57 aspirations are opt-in procedures. Participants can choose to undergo any number of aspirates: from zero to four

^l^Hemoglobin A1c will be assessed as part of CBC on screening day only

^m^Negative pregnancy test from day 0 will be confirmed prior to vaccination. Pregnancy testing will only be performed on days − 59 to − 14, 15, 28, and 57 if participants who can get pregnant undergo lymph node aspiration. Pregnancy test will be performed as part of the acute care panel, and additional volume is not required

^n^Antibodies against DENV1-4 will be assessed in all individuals. Flavivirus antibodies may include yellow fever virus, West Nile virus, Japanese encephalitis virus, and Zika virus, among others. These will be assessed at screening if necessary to confirm vaccination, travel, and/or medical history

Optional high resolution ultrasound-guided lymph node fine needle aspirations (LN FNA) will occur pre-vaccination and at days 15, 28, and 57 targeting the draining lymph node with the largest cortex and closest to the axillary vessels of the ipsilateral arm [39]. Ideally, at least five participants per group will undergo aspiration on days 15 and 28 at minimum. Pre-vaccination baseline LN FNA will be offered to all participants, but day 57 aspiration will only be offered to those who complete day 15 and/or day 28 LN FNA. Participants who undergo LN FNA will be contacted within 24 to 48 h to assess for any procedure-related AEs.

### Blinding

Participants will not be randomized because the primary endpoints are safety and immunogenicity, and the groups are defined by pre-existing immunity. To avoid biased assessment of AEs and immunogenicity markers, all study staff except the statistician and one computational biologist will be blinded to the final group assignment of each participant. To narrow the screening pool to potentially flavivirus-seronegative and dengue-seropositive individuals, all participants will receive a short, pre-screening Research Electronic Data Capture (REDCap) survey with questions about travel, residential, and flavivirus exposure history. These will be reviewed by the unblinded study staff, who will recommend individuals for screening to help meet the goal of 15 per group. Unblinded study staff will also provide the laboratory with sample identifying codes that require screening for other flaviviruses.

To maintain blinding, the clinical team will keep a password-protected list of participant names with corresponding sample identifying codes, and this list will only be available to the clinical team and unblinded study staff. The laboratory team will receive anonymized samples, generate and interpret the screening antibody data, and create a password-protected document that contains the sample identifying codes and group assignment. This document will only be available to the laboratory team and unblinded study staff. Throughout screening, these two documents will be shared with the statistician who will link participant names to group assignments and provide lists of individuals who are eligible for enrollment based on serology results only. All other eligibility criteria will be evaluated by the clinical team. Routine unblinding of group assignment will occur after all 45 participants have completed study day 57, which is the final day for primary and secondary endpoint assessments. AE assessments between days 58 and 180 are less likely to be influenced by baseline immune status, and immunogenicity assessments at days 90 and 180 focus on antibody waning, which is an exploratory endpoint.

### Safety run-in

Although we do not expect high viremia or SAEs in the heterotypic group, we will perform a safety run-in with the first six participants prior to the local mosquito season [40, 41]. This group will include two heterotypic participants since this is the group of highest concern. After the participants have completed their first 15 days, we will measure viremia via viral culture on samples collected at days 3, 6, 9, 12, and 15. Once we confirm that mean peak viremia titers remain $\le$10^3^ PFU/mL and no halting rules are met (Table 4), we will consent and vaccinate individuals ad-hoc and move to retrospective qRTPCR assessments of viremia. If any of the safety run-in participants has a mean peak viremia titer >10^3^ PFU/mL by culture, this would increase our concern for potential onward mosquito transmission, and we would limit vaccination to occur only outside of mosquito season. If any of the first participants has a mean peak viremia titer ≥10^6^ PFU/mL, we would halt the trial for safety concerns given this is the titer associated with symptomatic dengue.

**Table 4:** Halting Rules

• One or more participants experience an SAE that is possibly, probably, or definitely related to the study agent or research procedure (except for neutropenia – see specific guidelines below).
• Two or more participants experience the same or similar grade 3 or greater AEs that are unexpected and possibly, probably, or definitely related to a study agent. There are two exceptions to this rule:
◦ Neutropenia: halting rules are specific and listed below
◦ Local reactions to vaccine: The study will not be halted for any Grade 3 or lower AEs classified as local reactions to the vaccine.
• One or more of the first 4–6 participants experience a mean peak viremia titer of ≥ 10^6^ PFU/mL by viral culture.
• Two or more participants experience an ANC ≥ 500/µL but < 750/µL for > 5 days duration.^a^
• Two or more participants experience an ANC < 500/µL for any duration.^a^
• Two or more participants experience a vaccine-associated dengue-like syndrome, defined as infection^b^ associated with fever **and 2 or more** of the following symptoms:
◦ Grade 2 or greater headache lasting ≥ 12 h.
◦ Grade 2 or greater photophobia lasting ≥ 12 h.
◦ Grade 2 or greater generalized myalgia lasting ≥ 12 h.
• Any safety issue that the principal investigator or the CSO determines should halt the study. The DSMB may recommend a pause to the CSO.

^a^ These halting rules resulted from previous discussions between NIAID and the FDA on phase 1 clinical trials of rDEN3Δ30/31-7164 (reference IND 13,886)

^b^ Infection is defined as recovery of vaccine virus from the blood or serum of a participant and/or seropositivity or seroconversion to any dengue virus

### Clinical and laboratory procedures

Research blood collections will include serum at all study days, which will be used to assess viremia (days 3–15), cytokine signaling, antibody activity by GMTs and ELISAs, and antibody-dependent enhancement using enhancement assays with cell lines and primary monocytes [42]. Peripheral blood mononuclear cells (PBMCs) will be collected with pre-vaccination LN FNA and at days 0, 1, 3, 6, 9, 15, 28, 57, 90, 180, and 365. PBMCs will be characterized using a combination of multiparameter flow cytometry and single-cell sequencing. Analyses will include antibody panels for general cellular immunophenotyping, B and T cell activation status, and detection of antigen specific T cells through activation induced marker (AIM) assays. Intracellular cytokine staining will enable the detection of intracellular NS3 as a marker of DENV infection and replication [43, 44]. Human leukocyte antigen (HLA) typing will be performed on all individuals.

All LN FNA procedures will consist of 5 needle aspirations (passes) using a 25G bevel needle with a 10 mL syringe primed with 1 mL of air. Each pass will consist of 10 to 20 s of excursions using light suction. Each needle will be rinsed 2–3 times with 1 mL of media into one 15 mL collection tube, after which cells will be frozen and stored at -140ºC. All LN FNA will ideally be performed from the same lymph node via referencing or registration with local landmarks.

Through single cell immune profiling, paired gene and surface protein expression profiles (CITE-seq: Cellular Indexing of Transcriptomes and Epitopes by sequencing [45]), B and T cell receptor usage, and B cell antigen specificity (LIBRA-seq: linking BCR to antigen specificity through sequencing [46]) will be assessed pre-vaccination and at days 15, 28, and 57 for both peripheral blood and LN FNAs, with the potential to expand to earlier timepoints for PBMCs. We will use serotype-specific envelope protein probes to characterize the serotype-specific and cross-reactive B cell response after vaccination [46–48]. All sequencing, cellular, and antibody characterizations will be conducted retrospectively and in batches.

### Data collection, monitoring, and storage of research samples

All subjects will be a assigned a unique subject identifying code that will link subject name to biological samples. Study personnel involved in laboratory data acquisition, entry, and analysis will not have access to names, thereby protecting the privacy of subjects. Upon enrollment, participants will complete a comprehensive REDCap survey to collect data on time spent in dengue and flavivirus endemic areas, related febrile illnesses, and potential previous DENV infections. These will be reviewed by clinical staff after unblinding. Survey data will be stored in REDCap and clinical data will be stored in the Clinical Research Management Information System (CRIMSON). These secure, web-based applications are designed to support data capture for research studies and are the core data management systems used be NIAID. All data will be checked by study investigators, and external study monitors will verify that the trial is conducted and data are generated in compliance with the protocol, good clinical practice, and applicable regulatory requirements. All data will be archived at the end of the study and retained for the time period consistent with IRB requirements. All biological samples will be received, processed, aliquoted, and stored at NIAID and/or Fredrick National Laboratory. Final, anonymized laboratory data will be uploaded in ImmPort and the database of Genotypes and Phenotypes (dbGaP).

### Sample size justification

The sample size was chosen to power our primary safety (n = 45) and immunogenicity (n = 15 per group) endpoints. The primary safety endpoint is the frequency and severity of local and systemic reactogenicity signs and symptoms during the 28 day period after each vaccination, unexpected AEs up to 28 days after each vaccination, and SAEs through day 180 in any group (n = 45 total). The study will pause for a safety evaluation if one SAE or at least two similar ≥ grade 3 AEs that are possibly, probably, or definitely related to the vaccine are observed. Based on previous work with this vaccine candidate, we hypothesize that the true SAE probability is < 10%, and the probability of observing two similar grade 3 or 4 AEs is < 15%. If the true SAE rate is 10%, then there is a 90% probability that ≥ 1 SAE will be observed by the time 22 participants have been enrolled in the trial. Alternatively, if the true SAE rate is 0.2%, then there is a 10% probability of observing one SAE among 45 participants. If the true rate of ≥ 2 grade 3/4 AEs of similar types is 15%, then there is an 80% probability of observing this among the first 29 trial participants. However, if the true rate of 2 similar grade 3/4 AEs is 0.5%, then there is a 2.8% probability of observing this among the 45 participants.

The primary immunologic comparisons in this study are to determine whether there is an increase in DENV neutralizing antibody GMT between days 0 and 28 within each group and to compare mean peak viremia titer among groups measured from days 3 to 15. With 15 participants in each group, there will be 90% power to detect a fold-increase in GMT of 5.36 (or log_10_ = 0.73) assuming a standard deviation (SD) of 0.81 on the log_10_ scale using a 2-sided paired t-test with a significance level of 0.05. This SD is based on previous work, where authors assessed the change in DENV3 GMT between days 0 and 28 after tetravalent vaccination in DENV-seronegative and exposed groups [49]. In the exposed group, they reported that the change in GMT was 9 (log_10_ = 0.95) with a std of 0.81. In the seronegative group, the change in GMT was 15.6 (log_10_ = 1.19) with a std of 0.56. With regards to viremia, previous work reported a difference in the mean peak titer of 0.6 between a seronegative and seropositive group after monovalent DENV2 vaccination with SD of 0.19 in the seronegative group and 0.57 in the seropositive group [25]. With 15 per group, there will be 80% power to detect a difference of 0.55 if the SDs are 0.19 and 0.57 or a difference of 0.47 if SDs are the average of these two values at 0.38 using a 2-sided t-test with a significance level of 0.05/3 to compare all three groups. Therefore, this study is well powered to detect smaller differences than those reported in the literature.

### Statistical analysis

All participants who receive the single dose of vaccine will be analyzed for safety and immunogenicity. SAEs and AEs will be tabulated by grade and proportions of people with each AE. Comparisons of the SAEs and AEs among groups will be performed using Fisher’s exact for the overall test and Fisher’s exact unconditional tests and tests of proportions for two group comparisons (using R with fisher.test or uncondExact2 × 2 in exact2 × 2 library). All immunologic data will be analyzed using a log_10_ transformation. Observations below the lower limit of detection (LLOD) will have a value of ½ the LLOD imputed. Paired t-tests will be used to assess changes in continuous endpoints within each group, and t-tests will be used to assess changes in continuous endpoints among groups. Continuous data will be presented as means with standard deviations and confidence intervals. Regression models will also be used for continuous data to control for other variables. We plan to perform t-tests to compare continuous endpoints. However, we will examine the distribution of the data in a blinded fashion and determine whether, due to a skewed distribution, a Wilcoxon test will be more appropriate. Categorical data for immunologic endpoints will be presented as percentages with confidence intervals or relative risk or logistic regression models may be used to estimate risk or odds ratios. AE comparisons between groups will be performed at the 2-sided 0.05 level of significance. Comparisons of the primary and secondary immunogenicity endpoints will be performed at a 2-sided 0.05 level of significance. All tertiary comparisons will be considered exploratory and will be performed at a 2-sided 0.05 level of significance. As secondary analyses, regression models will also be performed to adjust for age, sex, gender, time since DENV exposure, and day 0 GMT. Exploratory analyses will include evaluating our immunophenotyping and multiomics data using stochastic mathematical models to contrast the mechanisms initiating and sustaining adaptive immune responses during primary infection with the immune pathways that are preexisting and recalled/reactivated during secondary and tertiary infections.

## Discussion

Natural infection cohort studies and vaccine clinical trials have revealed important patterns in host immune responses to DENV, but the mechanisms underlying cross-serotypic immunity and consistently accurate correlates of protection have not been identified. This trial will leverage a controlled environment and comprehensive immunologic techniques to directly compare how differing histories of natural DENV infection impact the host response to an attenuated DENV exposure. Most study participants will have a history of natural DENV infection, and some of these may have occurred years previously. Given that time from DENV exposure is associated with disease [6], and travel and migration are common in our globalized society, evaluating vaccine safety and immunogenicity in this population will inform vaccination efforts for travelers and expatriates. This trial will also expand our understanding of DENV3, which has never been modeled in sequential monovalent vaccination [36]. DENV3 is notable for its potential to undergo antibody-dependent enhancement, its unique impact on T cell responses with more of a focus on structural proteins compared to other serotypes, and its lack of a consistent correlate of protection [29, 32]. Specifically, in the TAK-003 vaccine trials, seronegative individuals were protected against DENV1 but not DENV3 despite similar antibody titers against both serotypes [50]. Comparing the immune responses to DENV3 in seronegative, heterotypic, and polytypic individuals may allow us to identify relevant markers and/or drivers of mild enhancement versus neutralizing immunity. Finally, evaluation of LN FNAs will allow us to assess how potent, cross-serotypic immunity develops and support the identification of novel mechanisms by which sequential exposure induces broadly protective immunity. By comparing viral kinetics and the B and T cell responses over time among the groups, we may identify more accurate markers associated with risk and protection.

This study has multiple strengths and weaknesses. The controlled environment with minimal risk for possible confounding natural infections will facilitate the exploration of basic questions in dengue immunology. Use of a full-length live attenuated DENV3 vaccine that induces B and T cell immune responses similar to those from natural infection facilitates the safe modeling of enhancement and protection [34, 51]. Recent work suggests that the mechanism of attenuation of the TV003 monovalent vaccine components is increased sensitivity of the virus strain to type I interferon signaling, and this form of attenuation is also observed in clinical isolates [52, 53]. Thus, markers associated with risk and protection will likely reflect those in natural infection. However, validation in endemic cohorts will be needed.

Trial limitations include the use of a single serotype, which may limit the generalizability of the immune responses to other serotypes. Recruitment, especially of the polytypic group, may be difficult in a non-endemic area. However, compared to the United States’ averages, Montgomery County, MD has a higher median income ($110,389 versus $65,712 in the United States) and number of non-native-born residents (32.1% vs. 13.7%) suggesting that there are likely more travelers and expatriates with previous DENV exposure in this area [54, 55]. Of the foreign-born Montgomery County residents, the top three most common countries of origin are all dengue endemic: 35.5% from El Salvador, 19.9% from India, and 12.9% from Guatemala. Additionally, our laboratory has a small observational cohort of local residents who have lived in or traveled to dengue endemic areas (NIH IRB# 11-I-0109). Despite limited recruitment effort, we have screened 33 individuals, enrolled 23, and identified 13 DENV-seropositive individuals. However, given the limited local recruitment pool, it may be difficult to match individuals by sex, age, and race/ethnicity. Thus, transcriptomic data may be influenced by demographic factors, and regression and machine learning modeling for high-dimensional data may be required for adjustments.

Finally, inclusion of the seronegative group is complex since monovalent vaccination will likely increase their risk of dengue with a future natural infection. However, since multiple vaccines have failed to successfully induce consistent, cross-serotypic protection in flavivirus-seronegative individuals, comparing immune responses in seronegative versus seropositive individuals is critical. Ideally, this work will inform more accurate assessments of future vaccine candidates in flavivirus-seronegative individuals. The risks to this group are expected to be the same as those in people who have been naturally infected with DENV. All groups will be advised to wear repellent and use precautionary measures such as screens when in DENV-endemic areas with high mosquito activity. Given the need to better protect flavivirus-seronegative individuals and the relatively straightforward methods that can be utilized to avoid severe dengue, we feel that inclusion of flavivirus-seronegative participants is justified. Our screening and consent process is very clear about this risk from first contact, and we will use comprehension quizzes to confirm understanding. We have also generated IRB approved letters for the participants and their primary care providers recommending that participants receive tetravalent dengue vaccination if one becomes available or if they relocate to an endemic area. If a dengue vaccine is approved for local residents within 5 years of study closure, we will attempt to contact all participants and recommend vaccination.

This study will simultaneously and extensively compare the immune responses after primary, secondary heterotypic, and tertiary or higher DENV exposures in naturally infected humans living in non-endemic areas. To our knowledge, this is the first study to sequentially assess LN FNA of draining nodes both before and serially post-DENV exposure, which should provide valuable immunologic data as has been demonstrated in HIV and SARS-CoV-2 [56, 57]. Further, this work aims to address two overarching questions in dengue literature. First, is dengue vaccination safe and immunogenic in individuals with distinct DENV exposure histories living in non-endemic areas? Second, by what mechanism does sequential exposure induce broadly protective dengue immunity? Collectively, this work may inform vaccine evaluation and design of new vaccine strategies to broaden potential target populations.

## Data Availability

Once the data have been collected and analyzed, results will be published in a peer-reviewed journal in PubMed and posted to clinicaltrials.gov. Sequencing data will be posted to the Database of Genotypes and Phenotypes (dbGaP). Results from other experiments including flow cytometry and ELISA assays will be posted to ImmPort Shared Data. Participants’ identity will remain anonymous and collected data will be presented in an aggregated form.
